# Dietary Diversity Score: Implications for Obesity Prevention and Nutrient Adequacy in Renal Transplant Recipients

**DOI:** 10.3390/ijerph17145083

**Published:** 2020-07-14

**Authors:** I-Hsin Lin, Tuyen Van Duong, Shih-Wei Nien, I-Hsin Tseng, Hsu-Han Wang, Yang-Jen Chiang, Chia-Yen Chen, Te-Chih Wong

**Affiliations:** 1 Department of Medical Nutrition Therapy, Chang Gung Memorial Hospital, Linkou 33305, Taiwan; cabbage@cgmh.org.tw (I.-H.L.); nina0904@cgmh.org.tw (S.-W.N.); cathy40422@cgmh.org.tw (I.-H.T.); 2 School of Nutrition and Health Sciences, College of Nutrition, Taipei Medical University, Taipei 11031, Taiwan; tvduong@tmu.edu.tw; 3 Department of Urology, Chang Gung Memorial Hospital, Linkou 33305, Taiwan; seanwang@cgmh.org.tw (H.-H.W.); zorro@cgmh.org.tw (Y.-J.C.); 4 Department of Nutrition and Health Sciences, Chinese Culture University, Taipei 11114, Taiwan; 209ru8u0438@gmail.com

**Keywords:** renal transplant recipients, obesity, dietary diversity, nutrient adequacy

## Abstract

Obesity affects both medical and surgical outcomes in renal transplant recipients (RTRs). Dietary diversity, an important component of a healthy diet, might be a useful nutritional strategy for monitoring patients with obesity. In this cross-sectional study, the data of 85 eligible RTRs were analyzed. Demographic data, routine laboratory data, and 3-day dietary data were collected. Participants were grouped into nonobesity and obesity groups based on body mass index (BMI) (for Asian adults, the cutoff point is 27 kg/m^2^). Dietary diversity score (DDS) was computed by estimating scores for the six food groups emphasized in the Food Guide. The mean age and BMI of participants were 49.7 ± 12.6 years and 24.0 ± 3.8 kg/m^2^, respectively. In the study population, 20.0% (n = 17) were obese. DDS was significantly lower in obese participants than in those who were not obese (1.53 ± 0.87 vs. 2.13 ± 0.98; *p* = 0.029). In addition, DDS was correlated with nutrition adequacy of the diet. Multivariate analysis showed that the odds of obesity decreased with each unit increase in DDS (odds ratio, 0.278; 95% confidence interval, 0.101–0.766; *p* = 0.013). We conclude that patients with higher dietary diversity have a lower prevalence of obesity.

## 1. Introduction

The prevalence of chronic kidney disease (CKD) in Taiwan is extremely high. Kidney transplantation is the best treatment for patients with end-stage of CKD [1]. However, obesity, which is abnormal or excessive body fat accumulation, is common in renal transplant recipients (RTRs). Up to 50% of RTRs experience weight gain after transplantation [2]. Findings derived from a national transplant database in United States revealed that the proportion of obese RTRs increased by 116% from 1987 to 2001 [3]. Obesity-related medical comorbidities and concerns regarding post-transplant outcomes were described in detail previously [4]. An analysis of more than 190,000 RTRs from the Scientific Registry of Transplant Recipients database revealed that obesity is associated with delayed graft function, graft failure, proteinuria, and acute rejection [5]. In addition, obesity is one of the risk factors for cardiovascular disease (CVD), hypertension, hyperlipidemia, insulin resistance and metabolic syndromes, which can lead to graft loss and the CVD-related mortality in RTRs [6,7,8,9]. Consequently, strategies designed to prevent post-transplant weight gain and other consequences associated with obesity in RTRs might improve the survival of patients and grafts.

Factors contributing to obesity during the post-transplant period have been well-researched [10]. Among these factors, inappropriate dietary intake, which is a major lifestyle-related risk factor for many chronic diseases, plays an important role in overweight and obesity among RTRs [10]. In general, RTRs have many dietary restrictions for a long time before transplantation. After successful kidney transplantation, improved renal function and appetite result in better nutrition status and advanced weight gain. In addition, an unrestricted and liberalized diet even leads to overnutrition problems in this population. Indices of dietary intake and dietary quality may be more informative for monitoring the overall diet and clinical outcomes in RTRs.

Few studies have investigated the well-established recommendation of healthy eating for RTRs, especially in Taiwan. In 2009, the Dietitians Association of Australia published guidelines for the nutritional management of adult RTRs, with particular focus on nutrient recommendations [11]. In our previous study [12], we observed that RTRs had inadequate intake of most nutrients, and that compliance with dietary recommendations was poor. The National Kidney Foundation (NKF) published a health guide for transplantation and recommended a healthful, balanced diet that includes foods from food guides, such as a variety of fresh fruits and vegetables, lean meats, reduced-fat dairy products, and whole grains, as well as a diet low in salt and high in fiber, for RTRs [13]. Studies investigating the connection between eating a balanced diet and clinical outcomes in RTRs are warranted.

Dietary diversity score (DDS), one of the indicators used for assessing dietary quality [14], is used to evaluate the diversity within food groups based on a healthful, balanced diet recommended in national food guides [15]. Studies have examined the association between DDS and obesity in the general population [14]; however, this index has rarely been employed in studies on RTRs. In this study, we hypothesize that DDS can be used as an independent determinant of obesity in RTRs.

## 2. Materials and Methods

### 2.1. Study Design and Settings

From September 2016 to June 2018, stable RTRs who were regularly followed up at the Department of Urology of Chang Gung Memorial Hospital were enrolled into this cross-sectional study. Participants were recruited through advertisements. Data regarding RTR demographics, anthropometric assessments, laboratory examinations, and dietary intake were collected by well-trained staff according to standardized methods and procedures. Moreover, participants were required to maintain their regular medications.

### 2.2. Patient Recruitment

**Figure 1:**
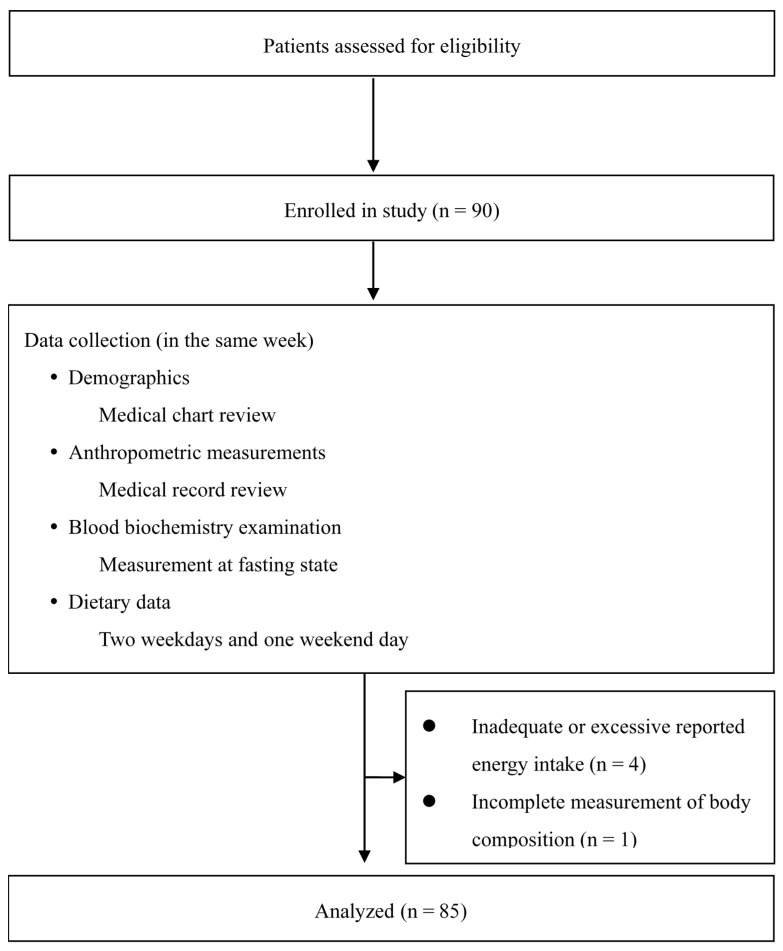
Flowchart of patient enrollment and study procedures.

Patients aged >18 years with stable graft kidney function who received maintenance immunosuppressive therapy (a calcineurin inhibitor-, antimetabolite-, and steroid-based regimen) were included. We checked urine albumin to creatinine ratio (ACR) for deterioration of graft kidney function. Prior to this study, the patients were informed of the purpose of the research. We excluded patients who had weight change >3 kg, acute rejection, glomerular filtration rate variation >25%, and change in immunosuppressive regimen 3 months prior to recruitment, as described previously [16]. Moreover, patients with malignant tumors, acute infection, history of dementia or cognitive impairment, inadequate or excessive reported energy intake, or missing assessment data were excluded. Finally, data of 85 RTRs were assessed. A flowchart of enrollment and study procedures is provided in Figure 1.

### 2.3. Demographic Data and Anthropometric Assessment

The demographic data collected included age, sex, height, and weight and were obtained from patients’ medical charts. We calculated body mass index (BMI) as weight (in kilograms) adjusted for the square of height (in meters). Patients were grouped into nonobesity and obesity groups according to the BMI cutoff point for obesity (BMI ≥ 27 kg/m^2^) suggested by the Taiwan Ministry of Health and Welfare (MOHW) [17]. Information on donor source, transplant vintage, and immunosuppressants was also collected through chart review.

### 2.4. Biochemical Parameters

Fasting blood samples were collected by registered nurses and were analyzed at the clinical laboratory using standardized methods. The following parameters were analyzed: albumin, blood urea nitrogen, creatinine, hemoglobin A1c, total cholesterol, triglycerides, high density lipoprotein-cholesterol, low density lipoprotein-cholesterol, high-sensitivity C-reactive protein, and insulin. The homeostatic model assessment of insulin resistance was used to assess insulin resistance, as described previously [18].

### 2.5. Dietary Data

All patients completed 3-day dietary records (2 weekdays and 1 weekend day) before they visited the well-trained dietitian at the latest follow-up. Moreover, 24-h dietary recall was completed through face-to-face interviews to confirm dietary records. Energy and nutrient intake was estimated using validated and reliable nutrient analysis software (COFIT Pro, Version 1.0.0; Cofit HealthCare Inc., Taipei, Taiwan) [19], which is based on the Taiwan MOHW Food and Drug Administration database [20]. The consumption of the six major food groups emphasized in Taiwan MOHW Food Guide (2017) was investigated: (1) whole grains and grain crops; (2) soybeans, fish, eggs, and meat; (3) vegetables; (4) fruits; (5) dairy products; and (6) oil and nuts.

### 2.6. Physical Activity

We used the short version of the International Physical Activity Questionnaire [21] to evaluate patients’ habitual physical activity levels. During the dietetic interview, patients self-described how much time (days per week and minutes per day) they spent performing physical activity of different intensity levels (vigorous, moderate, walking, and sitting). A metabolic equivalent (MET) value was calculated and is reported in MET-min/day, which has been previously described in detail [22,23].

### 2.7. DDS Calculation

We applied sex-specific serving sizes for each food group emphasized in the Taiwan food guide [24]. According to the guide, every Taiwanese adult should eat the following each day, based on the individual energy requirement: whole grains and grain crops (6–16 servings); soybeans, fish, eggs, and meat (3–8 servings); dairy products (1.5–2 servings); vegetables (3–5 servings); fruits (2–4 servings); and oil and nuts (4–8 servings). A score of 1 was given for participants who consumed at least one half-serving for each of the six food groups. The scores ranged from 0 to 6.

### 2.8. Nutrient Adequacy of the Diet: Nutrient Adequacy Ratio (NAR) and Mean Adequacy Ratio (MAR) 

The NAR of 14 nutrients was selected on the basis of Dietary Reference Intakes (DRIs) in Taiwan MOHW: vitamins B_1_, B_2_, B_6_, B_12_, A, C, and E; niacin; folic acid; calcium; magnesium; phosphorus; iron; and zinc [25]. NAR was calculated for each nutrient as the ratio of a participant’s daily intake to age- and sex-specific standard recommended amounts. MAR was calculated as the mean of the NAR of 14 nutrients. The formulas of NAR and MAR are presented below [26]:

$$\mathrm{NAR}=\frac{\mathrm{Daily}\;\mathrm{intake}\;\mathrm{of}\;\mathrm{nutrient}}{\mathrm{Age}-\;\mathrm{and}\;\mathrm{sex}-\mathrm{specific}\;\mathrm{standard}\;\mathrm{recommended}\;\mathrm{amounts}\;\mathrm{of}\;\mathrm{the}\;\mathrm{nutrient}}$$

$$\mathrm{MAR}=\frac{\mathrm{Sum}\;\mathrm{of}\;\mathrm{each}\;\mathrm{NAR}\;\mathrm{which}\;\mathrm{capped}\;\mathrm{at}\;1\;}{\mathrm{Nuber}\;\mathrm{of}\;\mathrm{nutrients}}$$

### 2.9. Ethical Considerations

All the standardized procedures used in this study were approved by the Chang Gung Medical Foundation Institutional Review Board (IRB201600954B0). All patients signed the informed consent form before the study was conducted.

### 2.10. Statistical Analysis

SAS version 9.4 software (SAS Institute, Cary, NC, USA) was used to perform all statistical analyses, and the significance level was set at *p* < 0.05. Data are presented as mean ± standard deviation, percentage, correlation coefficient, or odds ratio (OR) with 95% confidence interval (CI), as appropriate. The Shapiro–Wilk test and Q–Q plot were used to evaluate normality. Patients were divided into obesity and nonobesity groups, and the results were compared using Student’s *t* test and the Wilcoxon rank-sum test, as appropriate. We conducted multivariate logistic regression analysis adjusted for age, sex, total energy intake, and physical activity to identify independent risk factors for obesity, with DDS as the binary outcome variable.

## 3. Results

Table 1 summarizes the demographic, anthropometric, laboratory, and dietary characteristics of the study population. The mean age of participants was 49.72 ± 12.60 years, and 20.0% (n = 17) were obese. Among the 85 RTRs, the post-transplantation duration was 8.83 ± 5.97 years, and 82.4% (n = 70) of transplants were identified as originating from deceased donors. All patients were receiving maintenance immunosuppression regimens: 54 (63.5%) were receiving tacrolimus (FK506), and 31 (36.5%) were receiving cyclosporine.

The mean daily consumption of the six major food groups was as follows: whole grains and grain crops, 10.54 ± 2.69 servings; soybeans, fish, eggs, and meat, 5.81 ± 1.65 servings; dairy products, 0.19 ± 0.34 serving; vegetables, 2.50 ± 1.04 servings; fruits, 1.21 ± 1.02 serving; and oils and nuts, 10.0 ± 3.18 servings. As shown in Table 2, the mean DDS was 2.01 ± 0.98 in this study (range, 0–4). The maximum and minimum scores of diversity were found for oil and nuts (0.95 ± 0.21) and dairy products (0 ± 0), respectively.

Notably, the scores of diversity were positively correlated with total energy and macronutrient intake (*r*_energy_ = 0.59, *p* < 0.001; *r*_carbohydrate_ = 0.49, *p* < 0.001; *r*_protein_ = 0.66, *p* < 0.001; *r*_fat_ = 0.37, *p* = 0.001). Table 3 provides the correlation coefficients between DDS and NARs. A positive and significant correlation was found between DDS and most of the NARs as well as MAR.

### Comparison of Patients with and without Obesity

No statistically relevant differences were observed in age or total energy and macronutrient intake between the obesity and nonobesity groups (Table 1 and Table 2). However, obese patients had significantly lower DDS scores (1.5 ± 0.9 vs. 2.07 ± 1.0; *p* = 0.038). On the basis of suggestions in Taiwan’s food guide, none of our RTRs consumed sufficient dairy products daily. Moreover, compared with nonobese RTRs, obese RTRs consumed insufficient daily vegetables, had significantly higher BMI, and showed a worse lipid profile (high level of total triglycerides) and insulin resistance.

We examined the association between DDS and obesity using crude and multivariate-adjusted ORs with 95% CIs. With each unit increase in DDS, the crude odds of obesity significantly decreased by 49.5% (OR, 0.505; 95% CI, 0.275–0.928). This association remained significant after adjusting for age, sex, total energy intake, and physical activity (adjusted OR, 0.278; 95% CI, 0.101–0.766; *p* = 0.013).

## 4. Discussion

The present cross-sectional study showed that higher dietary diversity was significantly associated with 72% lower odds of obesity in RTRs. In addition, the results indicate that DDS is a good indicator of nutrition adequacy of diets (Table 3). We also observed that more nonobese than obese RTRs appeared to consume the recommended daily servings of each of the food groups, which was indicated as higher assigned scores in each component of DDS (Table 2). These results are more likely to be a direct/specific effect of such dietary quality on weight management in RTRs.

Previous studies show the high prevalence of obesity in RTRs. Johnson et al. observed that up to 50% of RTRs gained more than 10% body weight during the first year after kidney transplantation [27]. Armstrong et al. found that 21% of RTR had obesity after 7 years of transplantation [28]. Obesity is an ineffective prognostic factor in RTRs and may be involved in the adverse cardiovascular outcomes [28], progression of proteinuria, graft failure and acute rejection [5]. Appropriate dietary advice may enable the development of targeted strategies for treating obese RTRs.

DDS is a good indicator for assessing dietary quality in the general population [29,30,31] and diet-related health conditions, such as risk factors for cardiovascular disease [32], metabolic syndrome [33], and cause-specific mortality [34]. Our results suggest that monitoring dietary diversity based on food guides may be a useful method for evaluating the risk of obesity in RTRs. In the present study, an inverse association was observed between DDS and obesity, which is consistent with previous findings [35,36,37]. Oldewage-Theron and Egal asserted that adult women in South Africa had significantly higher BMI with lower DDS [35]. Azadbakht and Esmaillzadeh showed that Iranian female youth with higher DDS had lower abdominal adiposity and obesity [36]. Recently, Abris et al. also found a significant negative correlation between DDS and the prevalence of obesity among Filipino women [37]. According to our review of the relevant literature, many studies have investigated the association between DDS and obesity; however, studies focusing on patients with chronic kidney disease, particularly RTRs, are scant. Better dietary quality, as indicated by higher DDS, could have a profound influence on obesity prevention.

Other studies have provided controversial findings: there was a positive [38,39] or no [40,41] significant association between dietary diversity and obesity. Salehi-Abargouei et al. asserted that the inconsistency in these results can be explained as follows [14]: (1) food group variability defined by researchers to determine DDS; (2) different methods used for assessing dietary intake (e.g., food frequency questionnaire or dietary recall); (3) weighted scores based on the nutritional value or not; and (4) differences in numbers and characteristics of the study population, such as geographical differences or socioeconomic status. Our findings may not be generalizable to the entire transplant population. Further well-designed large-scale longitudinal studies using the similar approach of DDS to assess dietary quality are required to verify whether our observations can be extrapolated to other populations.

Another notable result of this study is that our obese RTRs seem not to have followed the recommendations in the food guide, which is indicated as lower scores for each component of DDS. In addition, the total scores of DDS were significantly lower in obese RTRs than in nonobese RTRs. Some evidence indicates an association between food guide adherence and overweight/obesity. So et al. reported that Canadians adults who followed Canada’s food guide recommendations, especially regarding the minimum servings of vegetables and fruits, had a lower prevalence of overweight/obesity and BMI [42]. A cross-sectional study from Australia also showed that low compliance with dietary guidelines was related to an approximately three-fold higher risk of being obese [43]. Our results showed that eating a variety and adequate amount of food, such as vegetables (which are low-energy density foods), does not add substantial energy to the overall caloric intake in the diet and results in favorable weight status.

In this study, positive correlations were found between DDS and nutrient adequacy for most micronutrients, implying that higher dietary diversity decreases the risk of micronutrient inadequacy. In addition, statistically positive relationships were identified between DDS and MAR (*r* = 0.50), consistent with the findings of studies conducted in Mali (*r* = 0.39) [44], the Philippines (*r* = 0.44) [45], and China (*r* = 0.37) [46]. NAR and MAR are indicators used to evaluate an individual’s daily intake of nutrients relative to the recommended intake. In this study, we used Taiwan DRIs for 14 nutrients as the reference values to calculate NAR. Our results provide detailed nutrient intake and quality information, showing that dietary compliance reflected by DDS corresponds to adequate nutrient intake. However, we found no relationship between DDS and nutrient adequacy for vitamins A, E, and C and calcium. The reasons for this result are unclear, but this finding implies that researchers assessing dietary quality should capture different dimensions together, such as dietary diversity and nutrient adequacy.

The present study also showed that few obese and nonobese RTRs consumed dairy products (Table 1), and that their intake of dairy products was inadequate; thus, the diversity scores of dairy products were 0 (Table 2). To the best of our knowledge, few studies have evaluated dietary intake in RTRs and have focused on food sources. Rho et al. found that Korean RTRs consumed less food derived from dairy products and had inadequate calcium intake [47]. This phenomenon is not surprising, because RTRs are required to avoid phosphorus-rich dairy products for a long time before kidney transplantation. In addition, the trend of lower dairy product consumption has been commonly found in Taiwan [48]. On the basis of NKF health guidance for transplantation [49], adult RTRs need approximately two servings of dairy products per day to maintain calcium and phosphorus levels. Education on a balanced diet and dietary consultation should be provided to RTRs.

Our study has several strengths and limitations. To the best of our knowledge, this is the first study to investigate associations between DDS and obesity in RTRs. The results should be interpreted cautiously because of the cross-sectional study design. Studies investigating dietary intake and trends of RTRs are scant, especially longitudinal studies. Further well-designed prospective studies and control trials are required to assess whether our observations can be extrapolated to other RTRs. In this study, we used 3-day dietary records, including workdays and off days, and a 24-h recall to determine dietary quality. These methods have been utilized to evaluate the dietary intake of RTRs and assess the nutrition-related problem [47]. In addition, we used individual-specific requirements for each food group, which may be more representative, to demonstrate the relationships of usual or habitual intake with adherence to dietary guidelines and the risk of obesity. However, different methods for assessing dietary intake and determining DDS may contribute to the inconsistent findings. Therefore, developing validated DDS as a dietary assessment tool is highly desirable. In the current study, we did not collect data on alcohol drinking habits, which are recognized as a major public health concern. Finally, our findings may still be limited by other unmeasured or residual confounding factors, such as the relatively small number of obese RTRs and the metabolic consequences of immunosuppressive agents.

## 5. Conclusions

We observed lower odds of obesity among RTRs consuming a balanced, diverse, and guideline-recommended diet. As DDS increased, the risk of micronutrient deficiency decreased. On the basis of evidence-based dietetic practice, the dietary recommendations for RTRs should emphasize adherence to dietary guidelines and increasing consumption of dairy products and vegetables for obesity prevention. Our study also introduced a novel strategy to alleviate obesity—the major complication after renal transplantation—which involves monitoring for the adequate intake of dietary food groups.

## Figures and Tables

**Table 1:** Demographic, anthropometric, clinical, and nutritional characteristics of the 85 renal transplant recipients ^1,2^.

	All (n = 85)			Nonobesity (n = 68)			Obesity (n = 17)		
**Demographics**									
Male/female	46/39			34/34			12/5		
Age, y	49.72 ± 12.60			50.4	±	12.84	47	±	11.54
Post-transplant, y	8.83	±	5.97	8.47	±	6.27	10.31	±	4.44
Tacrolimus/cyclosporine used	54/31			43/25			11/6		
Deceased/living donors	70/15			57/11			13/4		
**Anthropometry**									
Height, cm	161.39	±	8.61	160.87	±	8.73	163.47	±	8.01
Body weight, kg	62.88	±	13.26	58.84	±	10.51	79.06	±	10.59 *
BMI, kg/m^2^	24	±	3.83	22.64	±	2.86	29.46	±	1.84 *
**Laboratory**									
Albumin, g/dL	4.34	±	0.3	4.36	±	0.26	4.23	±	0.43
Blood urea nitrogen, mg/dL	24.05	±	11.59	24.38	±	12.5	22.75	±	6.97
Creatinine, mg/dL	1.43	±	0.76	1.42	±	0.8	1.45	±	0.61
Total cholesterol, mg/dL	208.2	±	45.34	210.18	±	48.34	200.29	±	30.46
Triglycerides, mg/dL	157.92	±	122.19	152.44	±	129.36	179.82	±	87.6 *
HDL-C, mg/dL	52.25	±	17.79	53.62	±	18.25	46.76	±	15.04
LDL-C, md/dL	121.45	±	37.59	121.74	±	40.51	120.29	±	23.31
HbA1c, %	6.06	±	1.01	6.05	±	1.09	6.11	±	0.55
Insulin, μU/mL	8.56	±	13.04	6.7	±	3.32	16.03	±	27.8 *
hs-CRP, mg/dL	5.16	±	12.2	5.13	±	13.26	5.29	±	6.68
**Dietary intake**									
Energy, kcal/day	1872.58	±	377.8	1857.59	±	354.7	1932.56	±	466.63
Carbohydrate, g/day	207.22	±	47.34	203.95	±	41.43	220.31	±	65.94
Protein, g/day	67.39	±	14.06	66.91	±	13.76	69.32	±	15.51
Fat, g/day	84.89	±	22.41	85.52	±	22.67	82.37	±	21.81
Whole grains and grain crops, servings	10.54	±	2.69	10.43	±	2.41	11.01	±	3.67
Soybeans, fish, eggs, and meat, servings	5.81	±	1.65	5.75	±	1.65	6.04	±	1.68
Vegetables, servings	2.5	±	1.04	2.56	±	1.09	2.27	±	0.81
Fruits, servings	1.21	±	1.02	1.19	±	1.01	1.3	±	1.08
Dairy products, servings	0.19	±	0.34	0.22	±	0.37	0.1	±	0.19
Oils and nuts, servings	10	±	3.18	10	±	3.28	10.02	±	2.85
**Others**									
eGFR, mL/min/1.73 m^2^	54.71	±	21.48	54.3	±	21.8	56.35	±	20.68
HOMA-IR	2.35	±	4.96	1.71	±	1.3	4.93	±	10.72 ^$^
Physical activity, MET-min/day	1034.66	±	414.57	944.86	±	347.04	1393.86	±	476.02 *

Abbreviations: BMI, body mass index; eGFR, estimated glomerular filtration rate; HbA1c, hemoglobin A1c; HOMA-IR, homoeostatic model assessment of insulin resistance; HDL-C, high density lipoprotein-cholesterol; hs-CRP, high-sensitivity C-reactive protein; LDL-C, low density lipoprotein-cholesterol; MET, metabolic equivalents. ^1^ Values are shown as the mean ± standard deviation or number, as appropriate. ^2^ Statistical analyses were conducted using Student’s *t* test or Wilcoxon rank-sum test, as appropriate. * *p* < 0.05; ^$^ *p* = 0.0593.

**Table 2:** Dietary diversity scores and diversity scores of food groups in obese and nonobese renal transplant recipients ^1,2^.

	All (n = 85)			Nonobesity (n = 68)			Obesity (n = 17)		
Whole grains and grain crops	0.24 ± 0.43			0.25	±	0.44	0.18	±	0.39
Soybeans, fish, eggs, and meat	0.54	±	0.5	0.59	±	0.5	0.35	±	0.49
Vegetables	0.15	±	0.36	0.19	±	0.4	0	±	0
Fruits	0.13	±	0.34	0.13	±	0.34	0.12	±	0.33
Dairy products	0	±	0	0	±	0	0	±	0
Oils and nuts	0.95	±	0.21	0.97	±	0.17	0.88	±	0.33
Total	2.01	±	0.98	2.13	±	0.98	1.53	±	0.87 *

^1^ Values are shown as mean ± standard deviation. ^2^ Statistical analyses were conducted using Student’s *t* test or Wilcoxon rank sum test, as appropriate. * *p* < 0.05.

**Table 3:** Correlation between dietary diversity score and nutrient adequacy for selected nutrients ^1^.

Nutrient Adequacy Of The Diet	Coefficient of Dietary Diversity Score	*p* Value
Vitamin A (RE)	0.15	0.1826
Vitamin B_1_	0.43	<0.0001
Vitamin B_2_	0.41	0.0001
Vitamin B_6_	0.49	<0.0001
Vitamin B_12_	0.28	0.0096
Vitamin C	0.09	0.4109
Vitamin E (α-TE)	0.08	0.4809
Niacin	0.50	<0.0001
Folic acid	0.38	0.0004
Iron	0.28	0.0118
Zinc	0.40	0.0002
Calcium	0.18	0.1121
Phosphorus	0.50	<0.0001
Magnesium	0.46	<0.0001
Mean adequacy ratio	0.50	<0.0001

Abbreviations: RE, retinol equivalent; α-TE, α-tocopherol equivalent. ^1^ Statistical analyses were conducted using Spearman’s rank correlation adjusted for age and sex.
